# Cucurbitane Triterpenoid from *Momordica charantia* Induces Apoptosis and Autophagy in Breast Cancer Cells, in Part, through Peroxisome Proliferator-Activated Receptor **γ** Activation

**DOI:** 10.1155/2013/935675

**Published:** 2013-06-13

**Authors:** Jing-Ru Weng, Li-Yuan Bai, Chang-Fang Chiu, Jing-Lan Hu, Shih-Jiuan Chiu, Chia-Yung Wu

**Affiliations:** ^1^Department of Biological Science and Technology, China Medical University, Taichung 40402, Taiwan; ^2^Division of Hematology and Oncology, Department of Internal Medicine, China Medical University Hospital, Taichung 40402, Taiwan; ^3^College of Medicine, China Medical University, Taichung 40402, Taiwan; ^4^Cancer Center, China Medical University Hospital, Taichung 40402, Taiwan; ^5^School of Pharmacy, College of Pharmacy, Taipei Medical University, Taipei 11031, Taiwan

## Abstract

Although the antitumor activity of the crude extract of wild bitter gourd (*Momordica charantia* L.) has been reported, its bioactive constituents and the underlying mechanism remain undefined. Here, we report that 3**β**,7**β**-dihydroxy-25-methoxycucurbita-5,23-diene-19-al (DMC), a cucurbitane-type triterpene isolated from wild bitter gourd, induced apoptotic death in breast cancer cells through peroxisome proliferator-activated receptor (PPAR) **γ** activation. Luciferase reporter assays indicated the ability of DMC to activate PPAR**γ**, and pharmacological inhibition of PPAR**γ** protected cells from DMC's antiproliferative effect. Western blot analysis indicated that DMC suppressed the expression of many PPAR**γ**-targeted signaling effectors, including cyclin D1, CDK6, Bcl-2, XIAP, cyclooxygenase-2, NF-**κ**B, and estrogen receptor **α**, and induced endoplasmic reticulum stress, as manifested by the induction of GADD153 and GRP78 expression. Moreover, DMC inhibited mTOR-p70S6K signaling through Akt downregulation and AMPK activation. The ability of DMC to activate AMPK in liver kinase (LK) B1-deficient MDA-MB-231 cells suggests that this activation was independent of LKB1-regulated cellular metabolic status. However, DMC induced a cytoprotective autophagy presumably through mTOR inhibition, which could be overcome by the cotreatment with the autophagy inhibitor chloroquine. Together, the ability of DMC to modulate multiple PPAR**γ**-targeted signaling pathways provides a mechanistic basis to account for the antitumor activity of wild bitter gourd.

## 1. Introduction

In Asia, bitter gourd (*Momordica charantia* L.) is widely used as a functional food to prevent and treat diabetes and associated complications [1]. In addition to the hypoglycemic effect, the antitumor activity of crude bitter gourd extract has also been reported in various types of cancer cells *in vitro* and *in vivo* [2–5]. Reported chemical constituents of bitter gourd include, but are not limited to, glycosides, saponins, alkaloids, fixed oils, triterpenes, polypeptides, and steroids (for review, see [6]). Wild bitter gourd, a wild species of *M. charantia* L., (*M. charantia* Linn. var. *abbreviate*), is native to several tropical areas of Asia, including southern Taiwan, where it is consumed not only as a vegetable but also as a herbal medicine. Recent studies have demonstrated that wild bitter gourd extracts exhibited multiple pharmacological activities associated with anti-inflammation and antidiabetics, including those of suppressing inflammatory response in macrophages [7], overcoming insulin resistance in skeletal muscle in fructose-fed rats [8], enhancing insulin signaling in skeletal muscle in high-fat-diet-fed mice [9], and upregulating mRNA expression of peroxisome proliferator-activated receptor (PPAR) *α*, PPAR*γ*, and their target genes in mice [10]. Together, these activities might account for the ability of wild bitter gourd to improve metabolic syndrome in humans [11]. 

PPAR*γ* regulates the expression of genes involved in the control of lipid metabolism and insulin sensitivity via the ligand-activated transcriptional activity [12–15]. PPAR*γ* ligands include naturally occurring fatty acids, 15-deoxy-delta12,14-prostaglandin J2 (PGJ2), and thiazolidinediones (TZDs), such as troglitazone and rosiglitazone [15]. Many of these PPAR*γ* agonists exhibit antiproliferative activities against many types of cancer cells including those of colon, prostate, and breast, suggesting the potential use of these agents in cancer therapy or prevention [16]. Evidence suggests that PPAR*γ* activation leads to the transcriptional suppression of a series of signaling effectors associated with tumorigenesis, including cell cycle regulators (cyclin D1, cyclin E, and cyclin-dependent kinase (CDK) 6) [17], antiapoptotic proteins (Bcl-2 and XIAP) [18], and cyclooxygenase- (COX-) 2 [19], thereby facilitating apoptotic death in cancer cells [20, 21]. Therefore, PPAR*γ* is recognized as a therapeutically relevant target for cancer therapy [22, 23].

**Figure 1:**
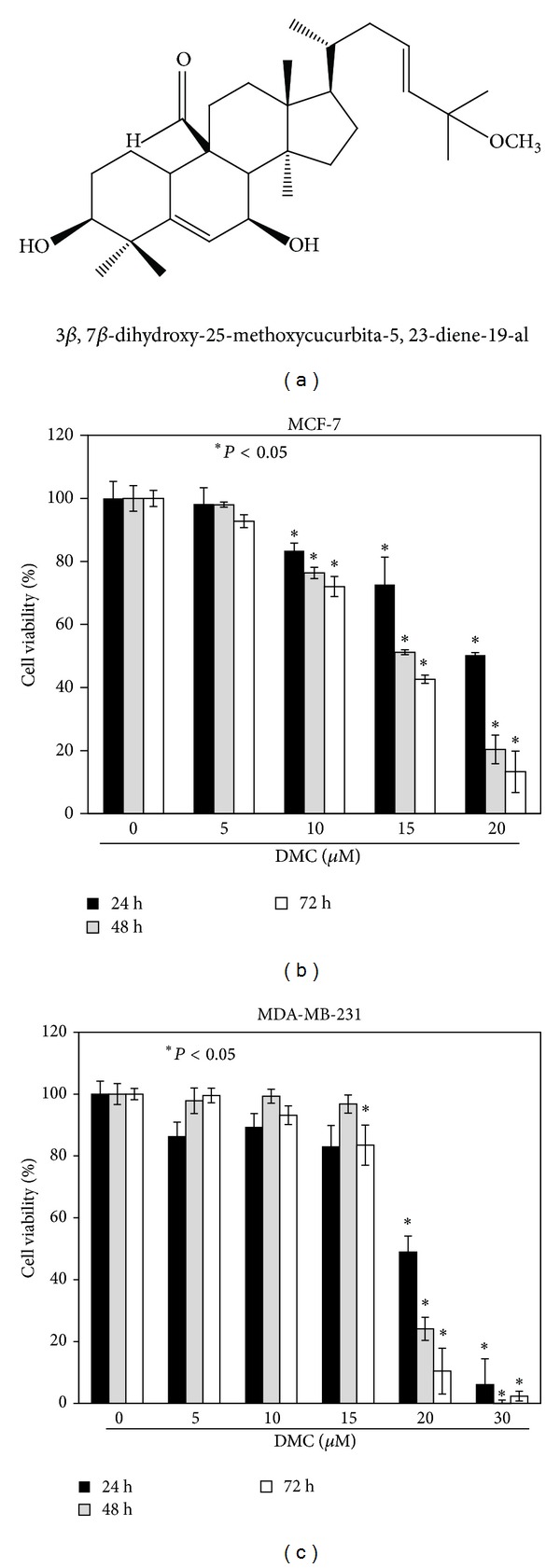
Antiproliferative effects of DMC in breast cancer cells. (a) The chemical structure of 3*β*,7*β*-dihydroxy-25-methoxycucurbita-5,23-diene-19-al (DMC). (b) Dose- and time-dependent suppressive effects of DMC on the viability of MCF-7 breast cancer cells. (c) Dose- and Time-dependent suppressive effects of DMC on the viability of MDA-MB-231 breast cancer cells. Cells were treated with DMC at indicated concentrations in 5% FBS-supplemented DMEM/F12 medium for 24, 48, and 72 h, and cell viability was determined by MTT assays. *Points*, mean; *bars*, SD (*n* = 6). *P < 0.05.

Although the health benefits of wild bitter gourd have been associated with PPAR*γ* activation [10], bioactive constituents that contribute to this pharmacological effect, however, remain undefined. In this study, we investigated the mechanism underlying the antitumor effect of 3*β*,7*β*-dihydroxy-25-methoxycucurbita-5,23-diene-19-al (DMC; structure, Figure 1(a)), a cucurbitane triterpenoid isolated from wild bitter gourd with antileukemic activity [24]. We obtained first evidence that DMC induced apoptotic death in breast cancer cells, at least in part, through a PPAR*γ*-dependent mechanism.

## 2. Materials and Methods

### 2.1. Plant Materials

DMC was isolated from the whole plant of *M. charantia* Linn. var. *abbreviate* collected in Pingtung County, Taiwan, in October 2008, and a voucher specimen (2008) has been deposited in the Department of Biological Science and Technology, China Medical University (Taichung, Taiwan). The identity and purity of purified DMC were verified by proton nuclear magnetic resonance (NMR) spectroscopy, high-resolution mass spectrometry, and 2D NMR spectrometry (Supplementary Material available online at http://dx.doi.org/10.115/2013/935675) using reported spectral data [25]. For *in vitro* experiments, DMC was dissolved in DMSO and was added to culture medium with a final DMSO concentration of less than 0.1%. Rabbit polyclonal antibodies against various biomarkers were obtained from the following sources: p-^473^Ser Akt, p-^308^Thr Akt, PARP, caspase-9, cyclin-dependent kinase (CDK) 6, p-^2448^Ser mTOR, mTOR, p-^216^Ser p70S6K, p-^79^Ser acetyl-CoA carboxylase (ACC), ACC, p70S6K, LC3, Atg7, GADD153, XIAP, GRP78, COX-2, Beclin 1, cyclin D1, p-^172^Thr adenosine monophosphate protein kinase (AMPK), AMPK, ER*α*, HIF1*α*, and NF-*κ*B (Cell Signaling Technologies, Beverly, MA, USA); Akt and Bcl-2 (Santa Cruz Biotechnology, Santa Cruz, CA, USA); *β*-actin (Sigma-Aldrich, St. Louis, MO, USA). The enhanced chemiluminescence (ECL) system for detection of immunoblotted proteins was from GE Healthcare Bioscience (Piscataway, NJ, USA). The GFP-LC3 and peroxisome proliferator-activated receptor response element (PPRE) x3-TK-Luc plasmids were kindly provided by Dr. Ching-Shih Chen at The Ohio State University. GW9662, chloroquine, and other chemical and biochemical reagents were obtained from Sigma-Aldrich unless otherwise is mentioned.

### 2.2. Cell Culture

MCF-7 and MDA-MB-231 human breast cancer cells were purchased from the American Type Culture Collection (Manasas, VA, USA). Cells were cultured in DMEM/F12 medium supplemented with 10% fetal bovine serum (FBS) (Gibco, Grand Island, NY, USA), 5 mg/mL of penicillin, 10 mg/mL of neomycin, and 5 mg/mL streptomycin at 37°C in a humidified incubator containing 5% CO_2_.

### 2.3. Cell Viability Analysis

 Effect of test agents on cell viability was assessed by using the 3-(4,5-dimethylthiazol-2-yl)-2,5-diphenyltetrazolium bromide (MTT) assays [26] in 6 replicates. Cells (5 × 10^3^) were seeded and incubated in 96-well, flat-bottomed plates in 10% FBS-supplemented DMEM/F12 for 24 h and were exposed to test agents at indicated concentrations for different time intervals. The medium was removed and replaced by 200 *μ*L of 0.5 mg/mL MTT in 5% FBS-DMEM/F12, and cells were incubated at 37°C for 2 h. Medium was removed and the reduced MTT dye was solubilized in 200 *μ*L/well DMSO. Absorbance was determined with a Synergy HT spectrophotometer (BioTek) at 570 nm.

### 2.4. Flow Cytometry

For assessment of apoptosis, 5 × 10^4^ cells were plated and treated with DMC at indicated concentration in 5% FBS-supplemented DMEM/F12 for 72 h. Cells were washed twice in ice-cold phosphate-buffered saline (PBS) and fixed in 70% cold ethanol for 4 h at 4°C, followed by spinning at 1200 rpm for 5 min and resuspending in ice-cold PBS containing 2% FBS. The cells were stained with Annexin V-FITC and propidium iodide according to the vendor's protocols (BD Pharmingen, San Diego, CA, USA) and analyzed by using BD FACSAria flow cytometer (Becton, Dickinson and Company). Caspase-3 activation was assessed using a FITC Rabbit Anti-Active Caspase-3 kit (BD Pharmingen) according the manufacturer's protocol.

### 2.5. Immunoblotting

Drug-treated cells were collected, washed with ice-cold PBS, and resuspended in lysis buffer, consisting of 20 mM Tris-HCl (pH 8), 137 mM NaCl, 1 mM CaCl_2_, 10% glycerol, 1% Nonidet P-40, 0.5% deoxycholate, 0.1% SDS, 100 *μ*M 4-(2-aminoethyl)benzenesulfonyl fluoride, leupeptin at 10 *μ*g/mL, and aprotinin at 10 *μ*g/mL. Soluble cell lysates were collected after centrifugation at 1500 ×g for 5 min, and equivalent amounts of protein (60–100 *μ*g) were resolved in 10% SDS-polyacrylamide gels. Bands were transferred to nitrocellulose membranes and blocked with 5% nonfat milk in PBS containing 0.1% Tween 20 (PBST) and incubated overnight with the corresponding primary antibody at 4°C. After washing with PBST three times, the membrane was incubated at room temperature for 1 h with the secondary antibody with PBST and visualized by enhanced chemiluminescence.

### 2.6. Transient Transfection

Plasmids were transiently transfected into cells by using the Fugene HD reagent (Roche) according to the manufacture's protocol. After 24 h, the transfected cells were treated with DMC or DMSO control and subjected to fluorescent analysis or Western blotting.

### 2.7. Confocal Imaging

MCF-7 cells expressing GFP-LC3 (2 × 10^5^/3 mL) were seeded in each well of a six-well plate and treated with DMC at the indicated concentration for 3 h. Cells were fixed in 2% paraformaldehyde (Merck) for 30 min at room temperature and permeabilized with 0.1% Triton X-100 for 20 min. Cells were washed with PBS and then subjected to examination on a Leica TCS SP2 confocal microscope (Leica Biosystems Nussloch GmbH, Heidelberg, Germany) examination.

For PPAR*γ* immunostaining, cells were fixed in 2% paraformaldehyde for 30 min at room temperature and permeabilized with 0.1% Triton X-100 for 20 min. After blocking with 1% bovine serum albumin (BSA), cells were incubated with mouse anti-human PPAR*γ* antibody at 4°C overnight, followed by anti-mouse IgG at room temperature for 1 h, washed with PBS, and subjected to fluorescent microscopic examination.

### 2.8. Transmission Electron Microscope

Cells were fixed in a solution containing 2% paraformaldehyde, 2.5% glutaraldehyde, and 0.2 M sodium cacodylate for 1 h. Fixed cells were suspended in a buffered solution containing 1% osmic acid for 1 h, followed by dehydration in a graded ethanol series, washing with acetone, and embedding into EPON epoxy resin. Ultrathin sections (60–80 nm) were prepared on an ultramicrotome and double-stained with uranyl acetate and lead citrate. All sections were examined and photographed with a Hitach H-600 transmission electron microscope.

### 2.9. Statistical Analysis

All data are presented as means ± SD obtained from three independent experiments. Statistical differences were calculated using Student's *t-*test, with the following symbols of significance level: *P < 0.05, **P < 0.01. 

## 3. Results and Discussion

### 3.1. Antiproliferative Effect of DMC in Breast Cancer Cells

The dose- and time-dependent effects of DMC on cell viability were assessed in 2 human breast cancer cell lines, ER*α*-positive MCF-7, and ER*α*-negative MDA-MB-231, by MTT assays (Figures 1(b) and 1(c)). Relatively, MCF-7 cells were more sensitive to the antiproliferative effect of DMC than MDA-MB-231 cells, with IC_50_ values of 14.3 and 17.6 *μ*M, respectively (Figures 1(b) and 1(c)). 

### 3.2. DMC Induces Apoptosis through Caspase Activation

**Figure 2:**
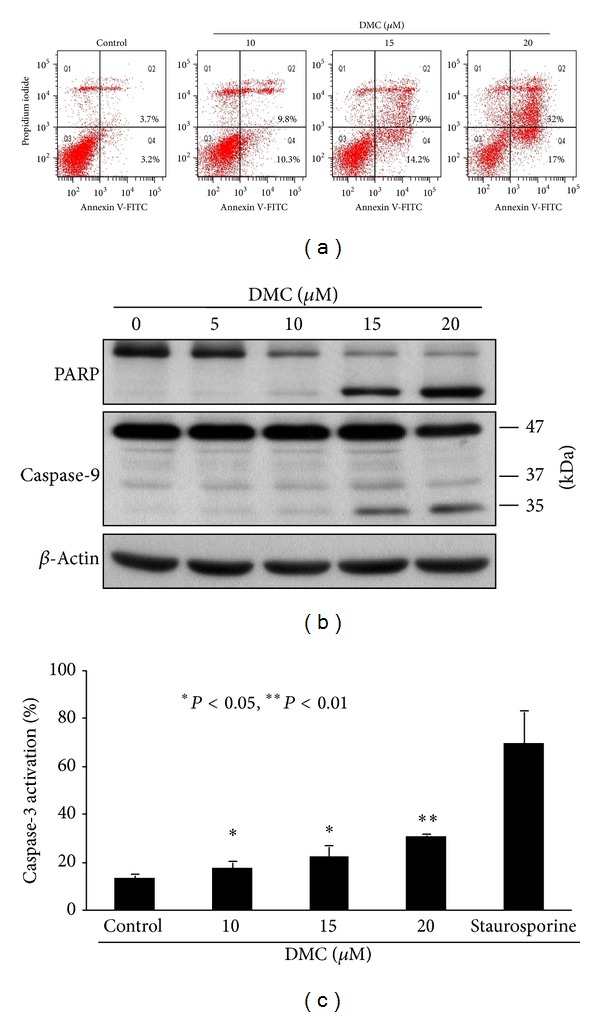
DMC induces apoptosis in MCF-7 cells. (a) Histograms showing the dose-dependent effect of DMC on Annexin V/PI staining. Cells were treated with DMC at indicated concentrations for 72 h, followed by Annexin V-PI staining. Data are representative of three independent experiments. (b) Dose-dependent effect of DMC on PARP cleavage and caspase-9 activation in MCF-7 cells after 72 h exposure in 5% FBS-supplemented DMEM/F12 medium. (c) Flow cytometry analysis of caspase-3 activity in MCF-7 cells after treatment with DMSO vehicle or the indicated concentrations of DMC for 72 h. 10 *μ*M staurosporine as the positive control. *P < 0.05, **P < 0.01.

We obtained several lines of evidence that DMC suppressed the proliferation of MCF-7 cells by inducing apoptosis. First, annexin V-propidium iodide staining reveals a dose-dependent increases in the proportion of apoptotic cells (defined as annexin V+ cells) after DMC treatment (Figure 2(a)). Second, Western blot analysis showed that DMC induced PARP cleavage and caspase-9 activation in a dose-dependent manner in MCF-7 cells (Figure 2(b)). Third, flow cytometric analysis shows a dose-dependent increase in the expression of activated caspase-3 in response to DMC (Figure 2(c)), confirming the involvement of caspase activation in DMC-mediated apoptosis. Treatment with DMC at 10, 15, and 20 *μ*M for 72 h increased the level of activated caspase-3, from 13% in the control group to 18%, 22%, and 30.5%, respectively (*P < 0.05 compared to the DMSO control). 

### 3.3. DMC Acts as a PPAR*γ* Agonist

**Figure 3:**
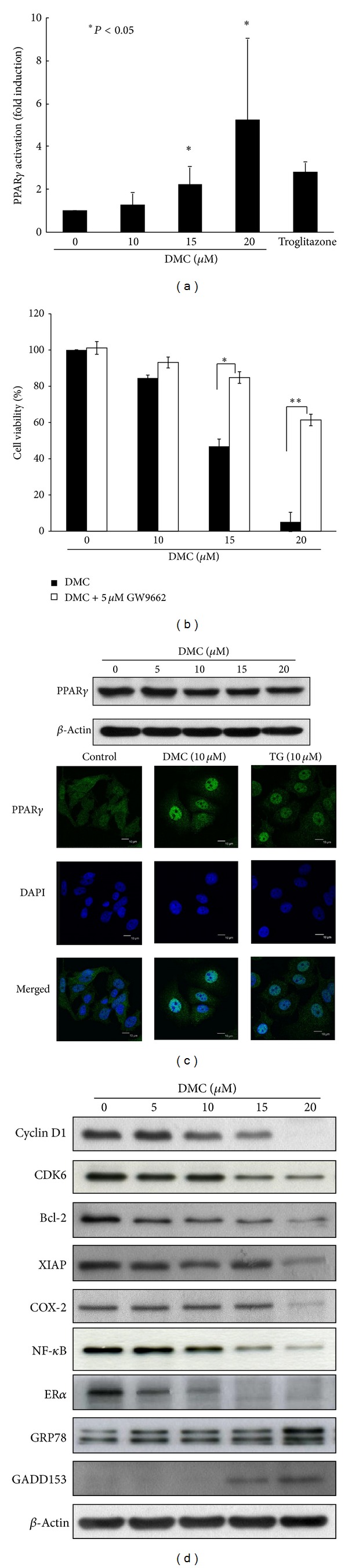
The PPAR*γ*-inducing activity of DMC. (a) Dose-dependent effect of DMC on PPAR*γ* activation in MCF-7 cells. Cells were transfected with PPRE x3-TK-Luc and Renilla plasmids for 24 h before treatment with DMC at indicated concentrations for 24 h. Data are expressed as percentage of the respective PPAR*γ* activity to the control. Normalized luciferase activity was determined and is shown as the fold activation relative result in DMSO-treated cells. Troglitazone at 50 *μ*M was used as positive control. Values are means ± S.E.M. of three independent experiments. *P < 0.05. (b) DMC-inhibited cell growth could be blocked by GW9662, an inhibitor of PPAR*γ*. Cells were treated with DMC at indicated concentrations in the presence of 5 *μ*M GW9662 or DMSO control for 72 h, and cell viability was determined by MTT assays. *Points*, mean; *bars*, SD (*n* = 6). *P < 0.05, **P < 0.01. (c) Effect of DMC on the protein expression and nuclear translocation of PPAR*γ*. MCF-7 cells were treated with DMC at the indicated concentrations for 72 h and detected PPAR*γ* by Western blotting (upper panel). MCF-7 cells were treated with 10 *μ*M DMC or 10 *μ*M troglitazone (TG) for 24 h, stained with anti-PPAR*γ*, and examined by confocal microscopy (lower panel). (d) Dose-dependent effect of DMC on the expression of various PPAR*γ*-targeted proteins in MCF-7 cells after 72 h exposure in 5% FBS-supplemented DMEM/F12 medium.

It has been reported that cucurbitane- and oleanane-type triterpenoids and saponins are potential active constituents of wild bitter gourd and that wild bitter gourd crude extracts activated PPAR*γ* signaling in mice [10]. We hypothesized that the antitumor activity of DMC was associated with its ability to facilitate PPAR*γ* activation. To corroborate this premise, MCF-7 cells were transiently transfected with PPRE-TK-Luc and Renilla plasmids and exposed to DMC at different concentrations or the positive control troglitazone at 50 *μ*M for 24 h. As shown, the PPRE-luciferase reporter assay indicates that DMC at 15 and 20 *μ*M increased the activity in PPAR*γ* transactivation by 2.2- and 5.2-fold, respectively (*P < 0.05), while troglitazone at 50 *μ*M gave rise to a 2.8-fold increase (Figure 3(a)). Equally important, cotreatment with GW9662, a PPAR*γ* inhibitor, protected cells from the suppressive effect of DMC on cell viability (Figure 3(b)). Although DMC did not alter the expression level of PPAR*γ* (Figure 3(c), upper panel), two lines of evidence support the effect of DMC on PPAR*γ* activation in MCF-7 cells. First, immunocytochemical analysis demonstrates the ability of DMC to facilitate the nuclear localization of PPAR*γ* in a manner similar to that of troglitazone (Figure 3(c), lower panel).

 Second, DMC was effective in modulating the expressions of various PPAR*γ*-targeted gene products associated with cell cycle progression and apoptosis in MCF-7 cells, including cyclin D1, CDK6 [17], Bcl-2, XIAP [18], and COX-2 [19] (Figure 3(d)). In addition, the drug effect on the expression of NF-*κ*B/p65 was interrogated in light of a recent report that PPAR*γ* acts as an E-3 ligase targeting p65 degradation [27]. As shown, DMC suppressed the expression of NF-*κ*B/p65 in a dose-dependent manner (Figure 3(d)). Moreover, the suppressive effect of DMC on estrogen receptor (ER) *α* expression is noteworthy (Figure 3(d)) as PGJ2 and TZD PPAR*γ* agonists have been shown to disrupt ER*α* signaling by facilitating ER*α* degradation [28]. 

Previously, it was reported that PGJ2 and thiazolidinedione PPAR*γ* agonists induce endoplasmic reticulum stress [29]. The ability of DMC to trigger this stress response was borne out by increased expression of two endoplasmic reticulum stress-associated markers, growth arrest- and DNA damage-inducible gene (GADD)153 (also known as CHOP), and GRP78 in MCF-7 cells (Figure 3(d)). GADD153 is a well-recognized endoplasmic reticulum stress-inducible transcription factor [30], and GRP78, a major endoplasmic reticulum chaperone, maintains endoplasmic reticulum integrity to mediate cell death when endoplasmic reticulum stress is beyond the tolerance of cell adaption [31]. 

### 3.4. DMC Induces Autophagy

**Figure 4:**
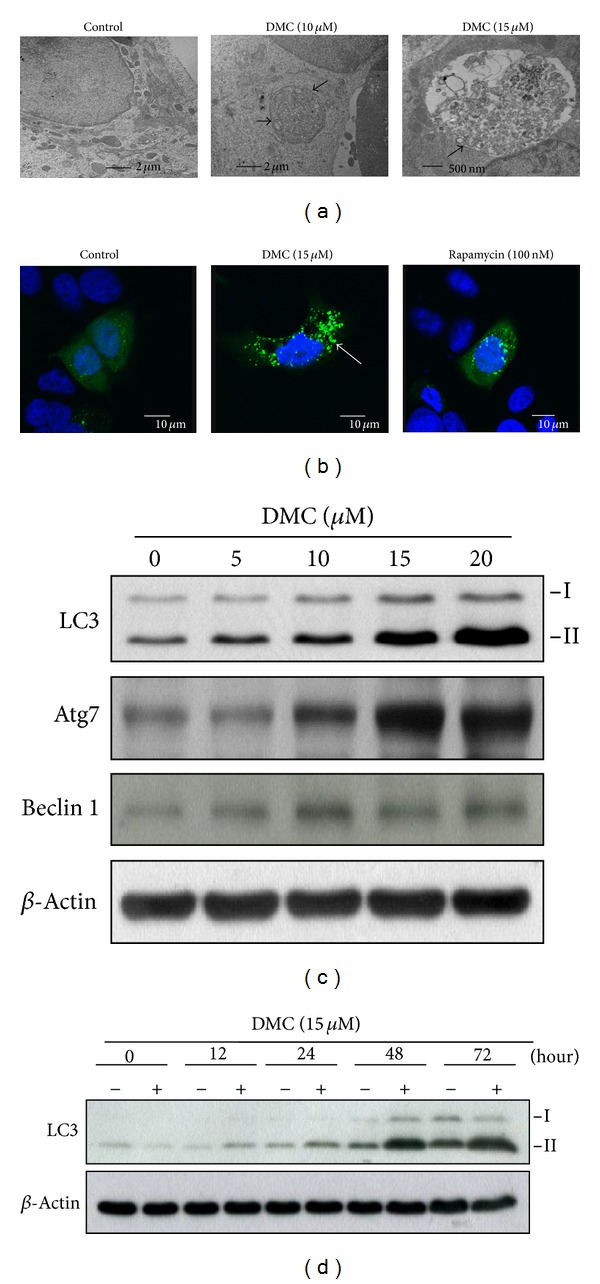
Induction of autophagy in MCF-7 cells by DMC. (a) Electron microscopic analysis of autophagosome formation in vehicle- or drug-treated MCF-7 cells as described in Section 2. Arrow: autophagosomes. (b) Fluorescent confocal microscopic analysis of drug-induced autophagosome formation in MCF-7 cells ectopically expressing GFP-LC3. The arrow indicates LC3-positive puncta in DMC-treated cells. MCF-7 cells transiently transfected with GFP-LC3 plasmids were treated with DMSO, 15 *μ*M DMC, or 100 nM rapamycin for 24 h and then fixed by 3.7% paraldehyde and examined by confocal microscopy. (c) Dose- and (d) time-dependent effects of DMC on the conversion of LC3-I to LC3-II and/or the expression of Atg7 and Beclin 1. MCF-7 cells were treated with DMC at indicated concentrations or DMSO for 72 h.

In light of a recent report that PPAR*γ* activation induces autophagy in breast cancer cells [15], we examined the effect of DMC on autophagy in MCF-7 cells. By using transmission electron microscopy, we found that exposure of MCF-7 cells to DMC at 10 or 15 *μ*M for 24 h led to autophagosome formation in the cytoplasm (Figure 4(a)). During autophagy, the cytoplasmic form of microtubule-associated protein 1 light-chain (LC3-I) is processed and recruited to the autophagosomes, where LC3-II is generated via site-specific lipidation. Thus, to confirm this drug-induced autophagosome formation, we assessed the effect of DMC on LC3-II conversion in two ways. First, MCF-7 cells were transiently transfected with GFP-tagged LC3 (GFP-LC3) and exposed to DMSO, 15 *μ*M DMC, or 100 nM rapamycin as a positive control. As observed by confocal fluorescence imaging, DMC induced the accumulation of LC3-positive puncta in the cytoplasm in a manner similar to that of rapamycin (Figure 4(b)). Second, Western blot analysis indicates that DMC treatment led to dose- and time-dependent increases in the abundance of endogenous LC3-II expression (Figures 4(c) and 4(d)). Moreover, our data show that this DMC-induced autophagy was associated with elevated expression levels of autophagy-related protein (Atg) 7 and Beclin 1 (a component of the class III phosphatidylinositol 3-kinase complex) (Figure 4(c)), both of which play a pivotal role in autophagy induction [32, 33]. 

### 3.5. DMC Targets mTOR Signaling via PPAR*γ* and AMPK Activation

**Figure 5:**
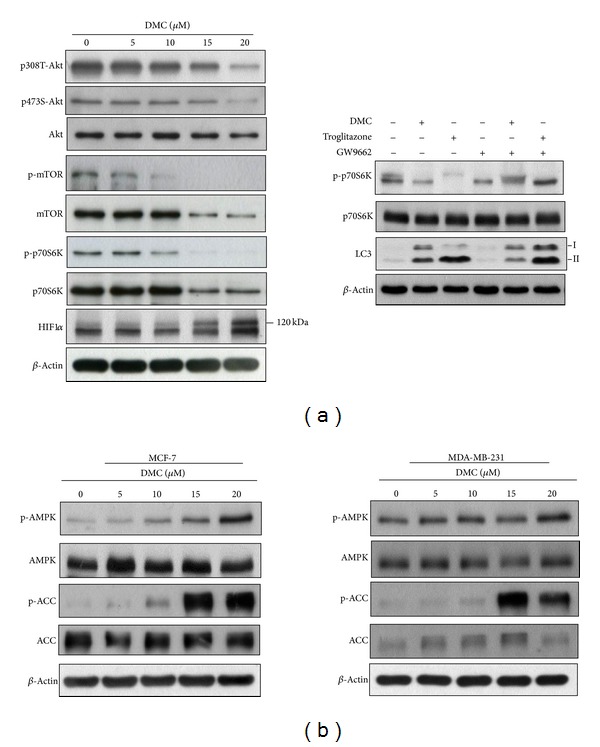
Effects of DMC on the activation status of the Akt-mTOR signaling axis and AMPK. (a) Dose-dependent of DMC on the phosphorylation/expression of Akt, mTOR, p70S6K, and HIF1*α* in MCF-7 cells after 72 h exposure in 5% FBS-DMEM/F12 (left panel). Effects of 15 *μ*M DMC, 15 *μ*M troglitazone, or 5 *μ*M GW9662 or the drug combination relative to DMSO control in MCF-7 cells after 72 h exposure in 5% FBS-DMEM/F12 (right panel). (b) Dose-dependent effects of DMC on the phosphorylation of AMPK and ACC in MCF-7 (left panel) and MDA-MB-231 (right panel) cells after 72 h exposure in 5% FBS-DMEM/F12.

Interruption of mTOR signaling is known to stimulate autophagy [34]. To investigate the mechanistic link between mTOR and DMC-induced autophagy, we assessed the activation status of the Akt/mTOR signaling axis by examining the phosphorylation status of Akt at Thr308, Ser473, and mTOR, and the mTOR target p70S6K in DMC-treated MCF-7 cells. As shown, DMC decreased the phosphorylation levels of all of these kinases in a dose-dependent manner (Figure 5(a), left panel). It was also reported that thiazolidinedione PPAR*γ* agonists induce autophagy through the activation of HIF1*α* [15], which led us to examine the expression of HIF1*α*. The result showed that DMC dose-dependently upregulated the expression of HIF1*α* (indicated by the 120 kDa band; Figure 5(a), left panel). To provide a mechanistic link among DMC-induced mTOR inhibition, PPAR*γ* activation, and autophagy, we examined the effect of the PPAR*γ* inhibitor GW9662 on these drug effects. Western blotting indicates that treatment of DMC or troglitazone, each at 15 *μ*M, decreased phosphorylation of p70S6K, indicative of mTOR inhibition, accompanied by increased accumulation of LC3-II as compared to the vehicle control (Figure 5(a), right panel). Although GW9662 alone had no appreciable effect on either cellular response, it was able to reduce the activities of DMC and troglitazone to induce p70S6K dephosphorylation and the conversion of LC3-I to LC3-II (Figure 5(a), right panel).

In addition, it is well recognized that AMPK activation promotes autophagy via mTOR inhibition [35]. Western blot analysis indicated that DMC facilitated concentration-dependent increases in the phosphorylation levels of AMPK and its downstream target acetyl-CoA carboxylase (ACC) in both MCF-7 and MDA-MB-231 cells (Figure 5(b)). The ability of DMC to activate AMPK in MDA-MB-231 cells is noteworthy as this cell line is deficient in liver kinase B1 (LKB1) [36], an upstream kinase of AMPK. Interestingly, DMC treatment led to a concentration-dependent increases in the expression level of ACC in MDA-MB-231 cells, which plateaued at 15 *μ*M, followed by a decrease at 20 *μ*M. This change, however, was cell line-specific as it was not noted in MCF-7 cells, of which the underlying mechanism remained unclear. 

Together, these findings suggest the ability of DMC to suppress mTOR signaling through the concerted action of Akt dephosphorylation and AMPK activation, which underlies the effect of DMC on autophagy induction.

### 3.6. Pharmacological Inhibition of Autophagy Enhances DMC-Induced Apoptotic Death

**Figure 6:**
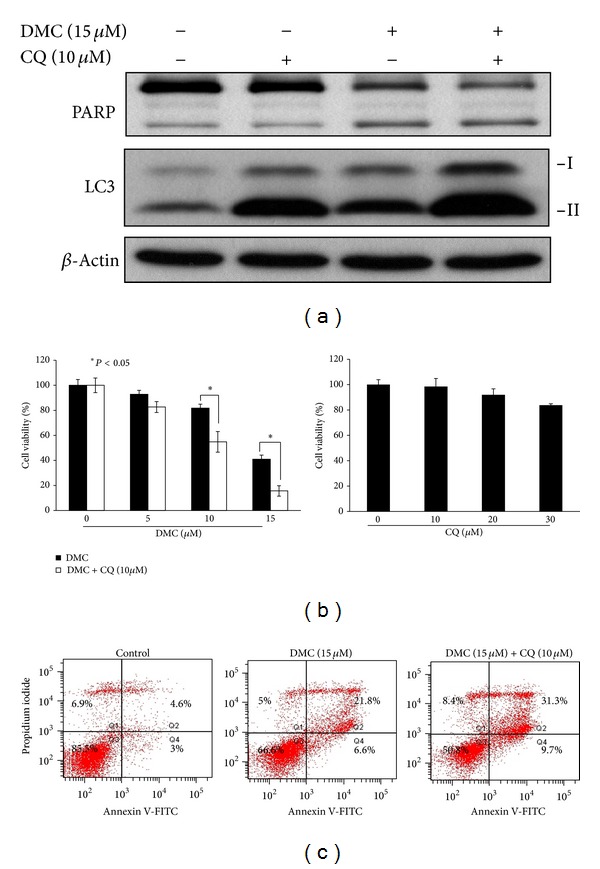
Effects of chloroquine (CQ) on DMC-induced autophagy and cytotoxicity. (a) Effects of 15 *μ*M DMC, 10 *μ*M chloroquine, or the drug combination relative to DMSO control in MCF-7 cells after 72 h exposure in 5% FBS-DMEM/F12. (b) MCF-7 cells were treated with chloroquine (CQ) alone (right panel) or in combination with DMC at indicated concentrations (left panel) for 72 h, and cell viability was determined by MTT assays. *Points*, mean; *bars*, SD (*n* = 6). *P < 0.05. (c) MCF-7 cells were treated with DMSO or DMC (15 *μ*M) alone or in combination with 10 *μ*M chloroquine (CQ) for 72 h exposure in 5% FBS-DMEM/F12, followed by Annexin V-propidium iodide staining. Data are representative of three independent experiments.

Autophagy has been reported to mediate a protective or enhancing effect on drug-induced cell death [37]. In light of the finding that autophagy acts as a survival signal in response to inhibitors of the PI3K-Akt-mTOR signaling axis [38], we examined the effect of chloroquine, an autophagy inhibitor, on DMC-mediated inhibition of MCF-7 cell proliferation. Chloroquine (CQ) inhibits autophagy at a later step in the pathway by blocking the fusion of autophagosome with lysosome and the subsequent lysosomal protein degradation [39]. As a consequence, treatment of CQ leads to increased LC3-II accumulation. As shown in Figure 6(a), exposure of MCF-7 cells to CQ, alone or in combination with DMC, showed a significantly higher accumulation of converted LC3-II relative to that of vehicle- or DMC-treated cells. This LC3-II accumulation is indicative of the inhibition of autophagy by CQ in MCF-7 cells. It is noteworthy that while the drug showed no antiproliferative activity against MCF-7 cells, chloroquine at 10 *μ*M significantly enhanced the suppressive effect of DMC on viability of MCF-7 cells (Figure 6(b)). Annexin V-staining and Western blot analyses suggest that this increase in cytotoxicity was attributable to increased apoptosis as a result of autophagy inhibition (Figures 6(a) and 6(c)).

## 4. Discussion

Wild bitter gourd has been reported to exert anti-inflammatory [7], antioxidant [40], and hypoglycemic effects [41] in various experimental animal models. However, its active constituents contributing to these pharmacological effects have not been fully characterized. In this study, we demonstrated that DMC, a cucurbitane-type triterpene isolated from wild bitter gourd, acts as a PPAR*γ* agonist. Although the activity of other cucurbitane-type triterpenes in modulating PPAR*γ* activity remains to be investigated, the ability of DMC to activate PPAR*γ* might underlie the hypoglycemic effect of wild bitter gourd crude extracts in diabetic mice [41]. 

 Several lines of evidence indicate that PPAR*γ* plays a pivotal role in mediating DMC's antiproliferative activity in breast cancer cells. First, inhibition of PPAR*γ* by the pharmacological inhibitor GW9662 protected cells from the inhibitory effect of DMC on the viability of MCF-7 cells (Figure 3(b)). Second, DMC suppressed the expression of a series of PPAR*γ*-targeted signaling effectors that govern cell cycle progression, proliferation, and survival, including cyclin D1, CDK6, Bcl-2, XIAP, COX-2, NF-*κ*B, and ER*α* (Figure 3(d)). Particularly, the downregulation of ER*α* expression is noteworthy since ER*α* has been shown to bind to PPRE and negatively interfere with PPAR*γ* signaling in breast cancer cells [42]. Thus, this ER*α*-ablating effect might account for the higher sensitivity of MCF-7 cells relative to MDA-MB-231 cells to DMC (Figure 1(b)). Third, DMC induced autophagy through mTOR inhibition (Figures 4 and 5), which is reminiscent with that reported with TZD PPAR*γ* agonists in breast cancer cells [15]. 

 mTOR, a central cell growth regulator that integrates growth factor and nutrient signals [43], is positively and negatively regulated by Akt and AMPK, respectively. This DMC-facilitated mTOR downregulation is likely attributable to the concerted action of DMC to facilitate Akt dephosphorylation and AMPK activation (Figure 5). The ability of DMC to activate AMPK is noteworthy as AMPK is a key energy sensor and regulates cellular metabolism to maintain energy homeostasis [44]. Previously, TZD PPAR*γ* agonists were reported to induce PPAR*γ*-independent AMPK activation through changes in cellular energy state [45, 46]. However, the finding that DMC was equally effective in activating AMPK in LKB1-deficient MDA-MB-231 and MCF-7 cells suggests that this activation might be independent of the cellular metabolic status as LKB1 is activated in response to environmental nutrient changes [47]. Thus, the mode of AMPK activation by DMC warrants further investigation. 

 The Akt/mTOR pathway is often dysregulated in malignant cells, thus representing an important target for cancer prevention and therapy [48]. The concurrent suppressive effect of DMC on Akt and mTOR activation is noteworthy, because it circumvents the feedback activation that results from mTOR inhibition, a problem associated with rapamycin-based mTOR inhibitors [49]. From a clinical perspective, the ability of DMC to concurrent target Akt and mTOR underlies its translational potential as a chemopreventive agent. Nonetheless, consistent with the reported effect of mTOR inhibitors and many other anticancer agents, DMC induced a cytoprotective autophagy that decreased DMC's antiproliferative potency, which, however, could be overcome by the co-treatment with the autophagy inhibitor chloroquine (Figure 6). Further *in vivo* studies are needed to better understand the role of DMC, alone or in combination with other agent, in breast cancer prevention and treatment.

## 5. Conclusion

There has been growing interest in the use of wild bitter gourd as a dietary supplement for the treatment of various illnesses in light of its diverse pharmacological effects, including hypoglycemic, anti-inflammatory, antibacterial, antiviral, and antitumor activities. However, despite recent advances in gaining understanding of the health beneficial effects of this herbal medicine, information regarding the mode of action of its bioactive ingredients is lacking or fragmentary. In this study, the activity of DMC isolated from wild bitter gourd in PPAR*γ* activation was characterized for the first time. As the role of PPAR*γ* in regulating lipid metabolism, insulin sensitivity, apoptosis, and cell differentiation is well recognized, the ability of DMC to modulate multiple PPAR*γ*-targeted signaling pathways provides a molecular basis to account for the hypoglycemic and antitumor activities of wild bitter gourd. Today, processed bitter gourd in the form of capsules or tablets is used by natural health practitioners, which like many other herbal medicine suffers from difficulty in quality control due to high variability of bioactive compounds involved. From a clinical perspective, wild bitter gourd extracts enriched in DMC and related compounds, of which the contents could be quantified by chromatographic fingerprint analysis, will provide a better alternative to capsules or tablets for disease management.

## Supplementary Material

1D, 2D NMR, and Mass spectra of 3beta,7beta-dihydroxy-25-methoxycucurbita-5, 23-diene-19-al (DMC).
