# Supplementary material for: Cucurbitane Triterpenoid from Momordica charantia Induces Apoptosis and Autophagy in Breast Cancer Cells, in Part, through Peroxisome Proliferator-Activated Receptor γ Activation
Source: Evid Based Complement Alternat Med. 2013 Jun 13;2013:935675. doi: 10.1155/2013/935675 (PMC3697288; doi:10.1155/2013/935675)
Supplement: Supplementary file 1 — 1D, 2D NMR, and Mass spectra of 3beta,7beta-dihydroxy-25-methoxycucurbita-5, 23-diene-19-al (DMC). [file 935675.f1.pdf]

SUPPORTING INFORMATION

Cucurbitane triterpenoid from *Momordica charantia* induces apoptosis and autophagy in breast cancer cells, in part, through peroxisome proliferator-activated receptor γ activation

Jing-Ru Weng[1,*], Li-Yuan Bai [2,3], Chang-Fang Chiu [2,4], Jing-Lan Hu[1], Shih-Jiuan Chiu[5], and Chia-Yung Wu[1]

[1]*Department of Biological Science and Technology, China Medical University, Taichung, 40402 Taiwan,* [2]*Division of Hematology and Oncology, Department of Internal Medicine;* [3]*Cancer Center, China Medical University Hospital, Taichung, 40402 Taiwan,* [4]*College of Medicine, China Medical University, Taichung, 40402 Taiwan,* [5]*School of Pharmacy, Taipei Medical University, Taipei, 11031, Taiwan*

Figure		Page
S1	^{1}H NMR spectrum (600 MHz, $CDCl_3$) of DMC	3
S2	^{13}C NMR spectrum (150 MHz, $CDCl_3$) of DMC	4
S3	HMQC spectrum of DMC	5
S4	COSY spectrum of DMC	6
S5	HMBC spectrum of DMC	7
S6	NOESY spectrum of DMC	8
S7	Mass spectrum of DMC	9

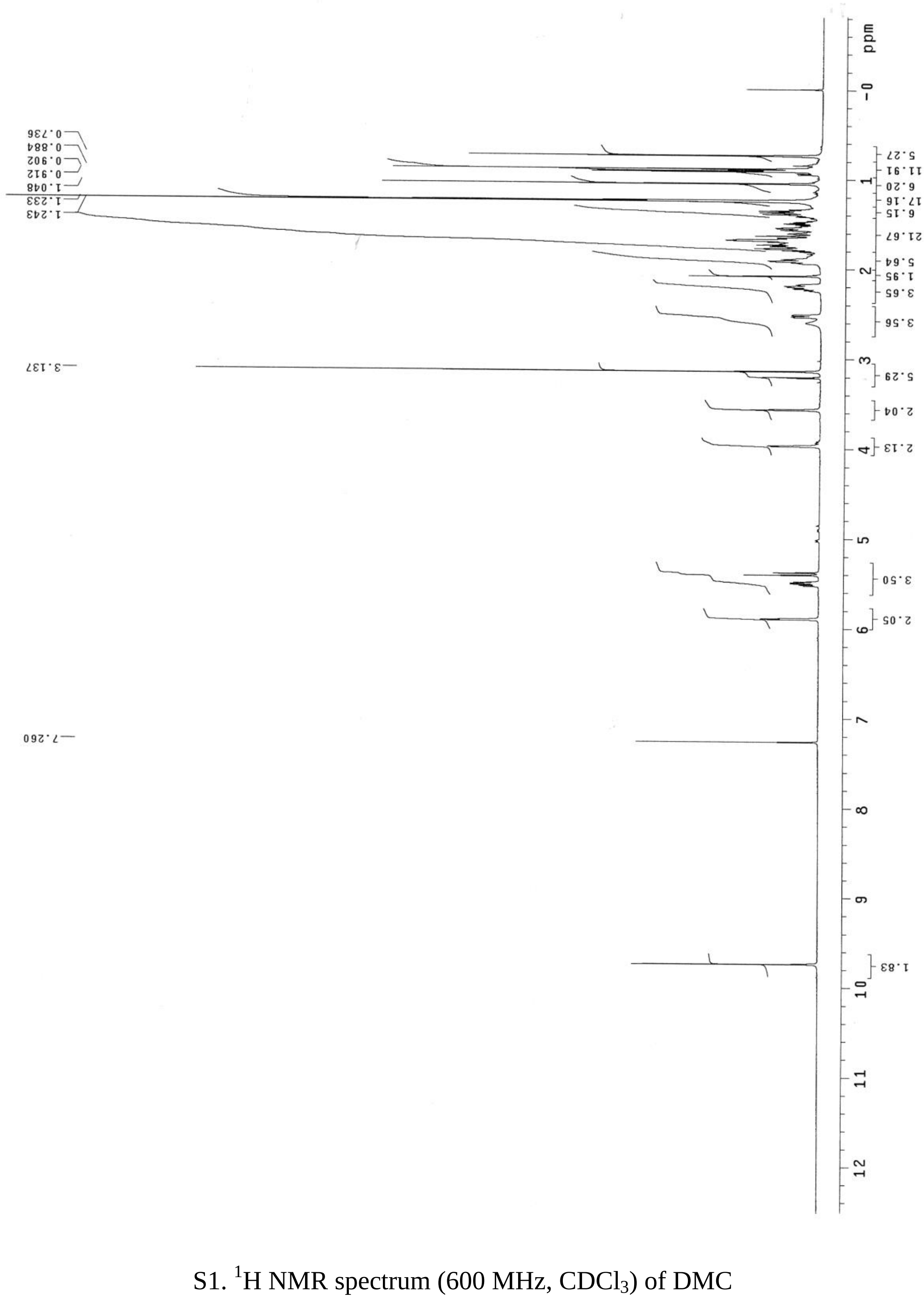

S1. ^{1}H NMR spectrum (600 MHz, $CDCl_3$) of DMC

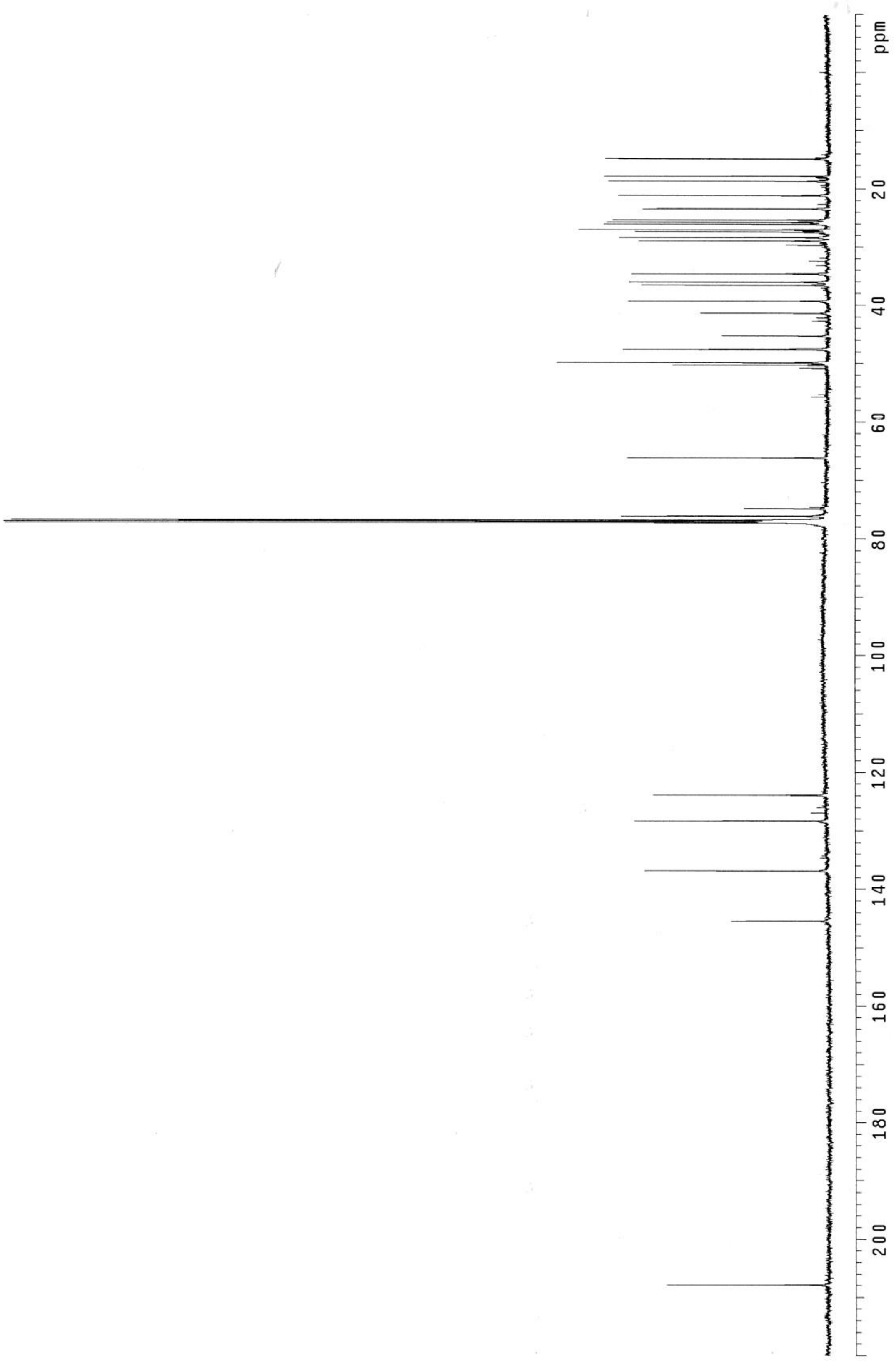

S2. ^{13}C NMR spectrum (150 MHz, $CDCl_3$) of DMC

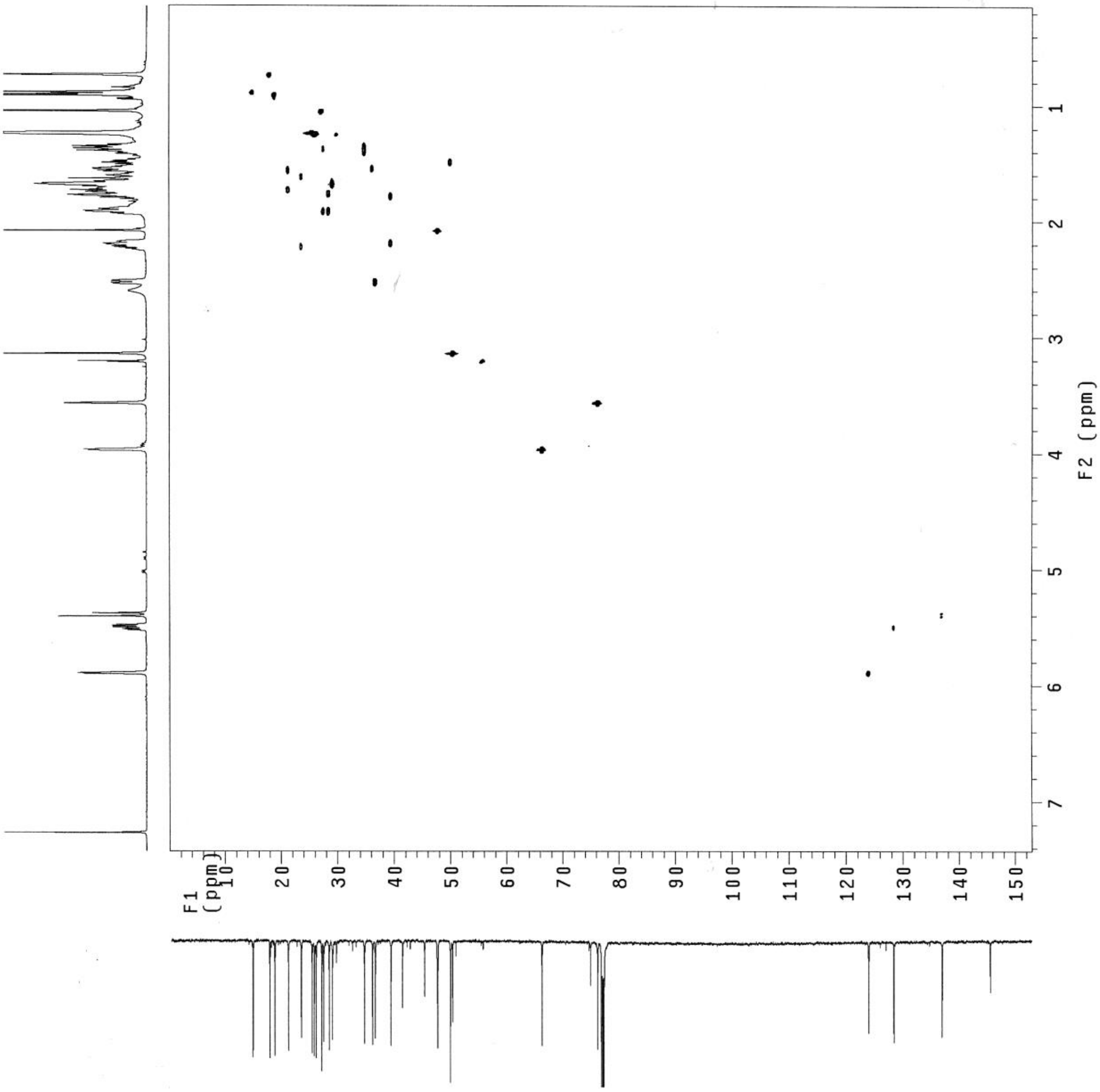

S3. HMQC spectrum of DMC

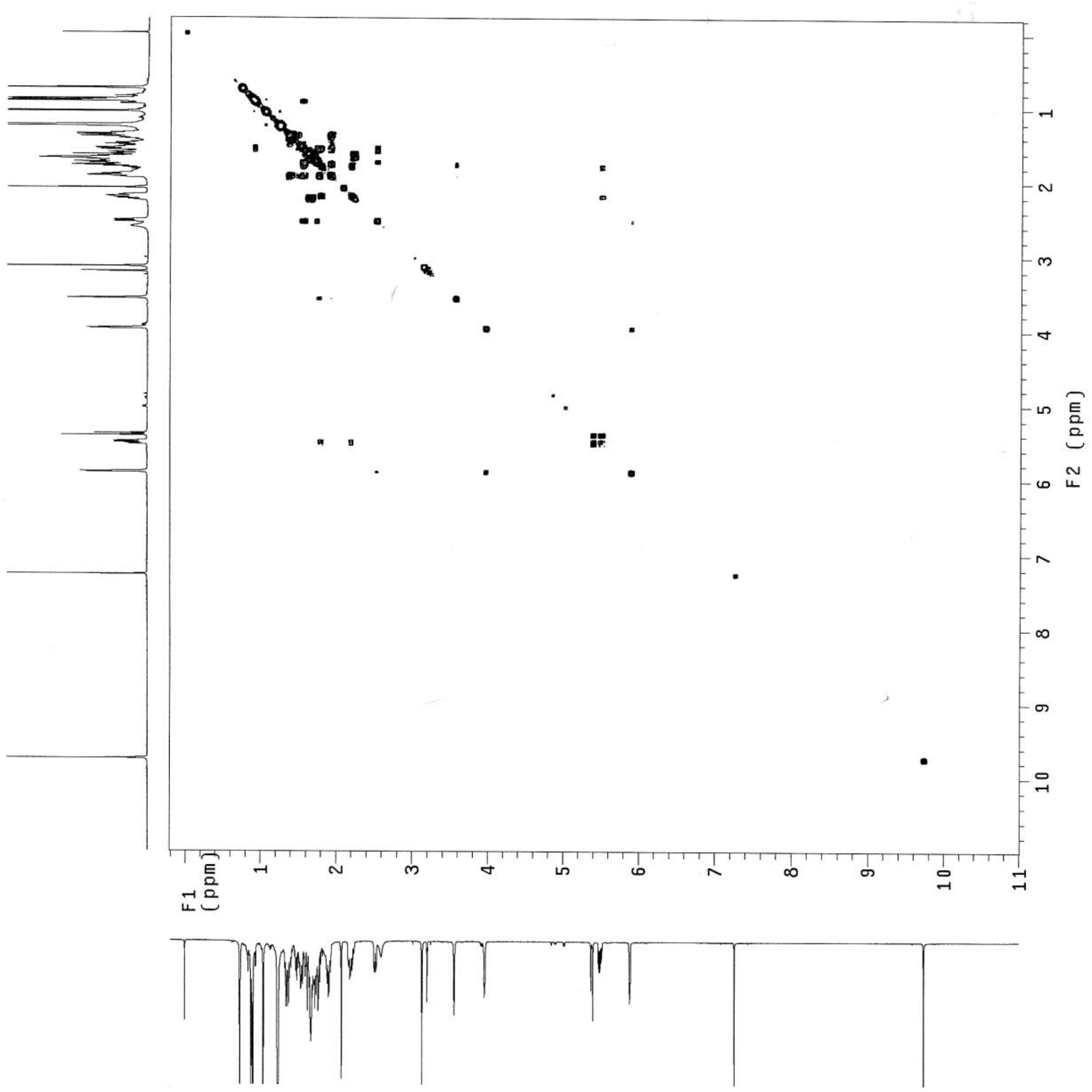

S4. COSY spectrum of DMC

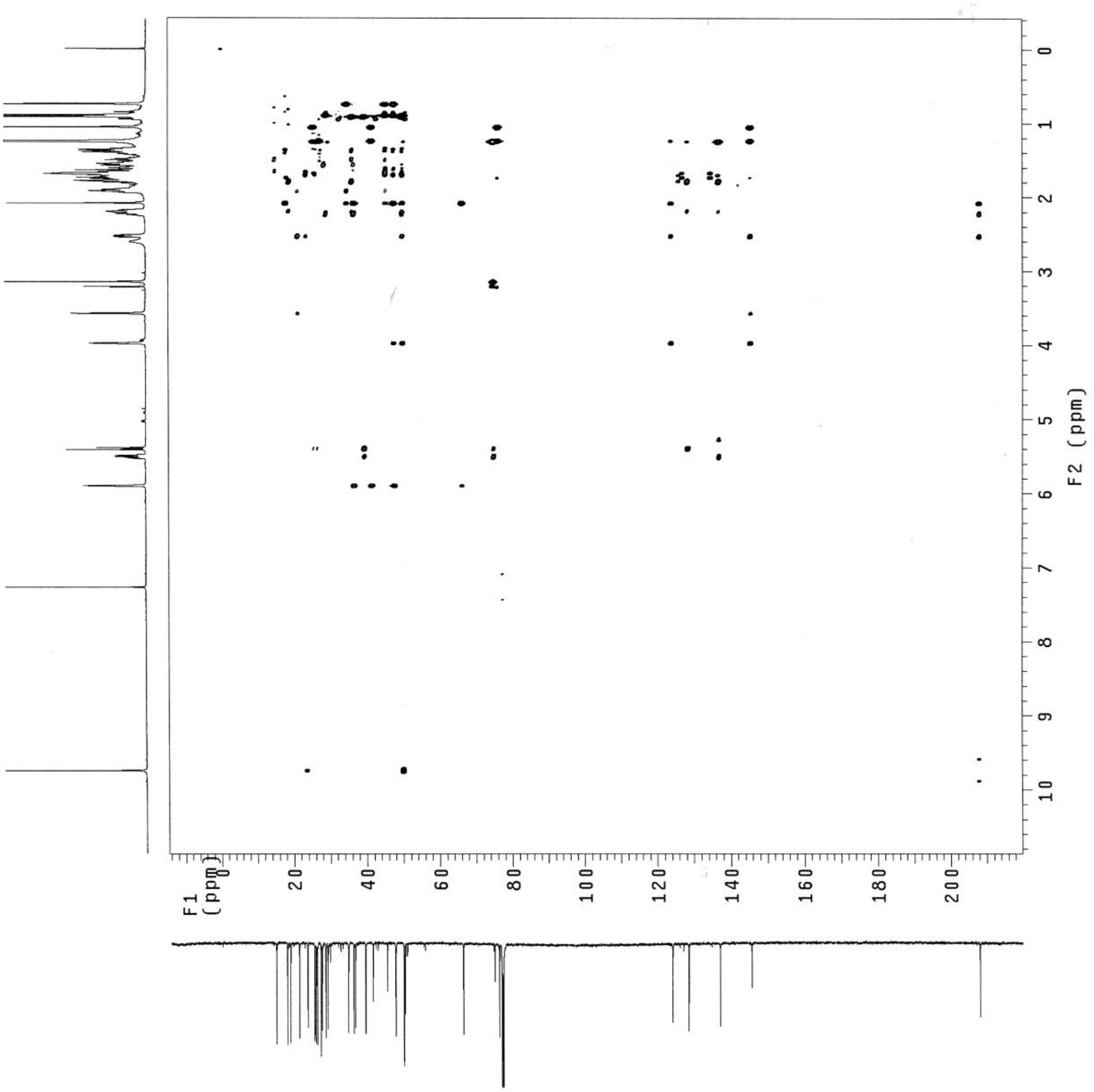

S5. HMBC spectrum of DMC

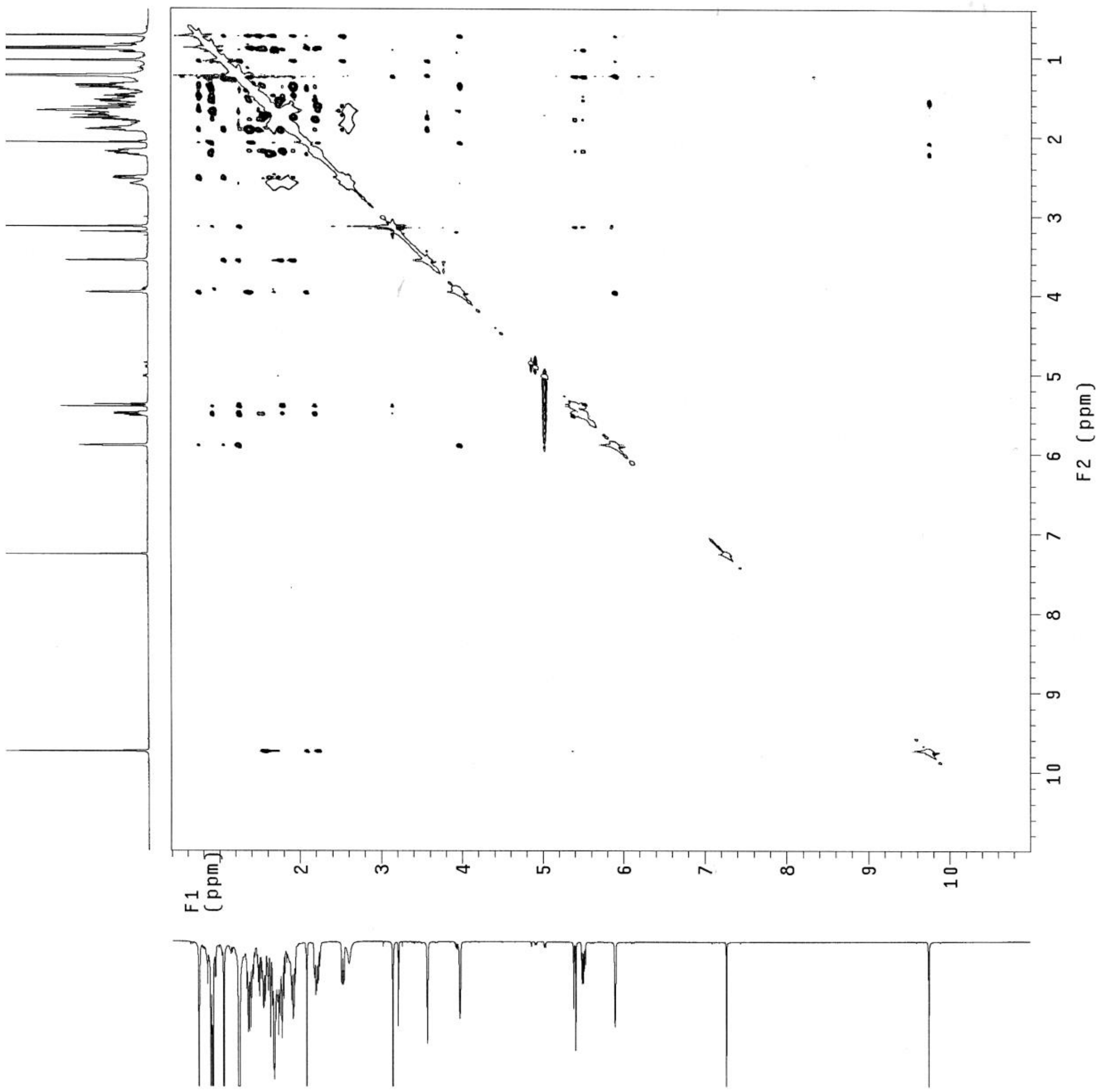

S6. NOESY spectrum of DMC

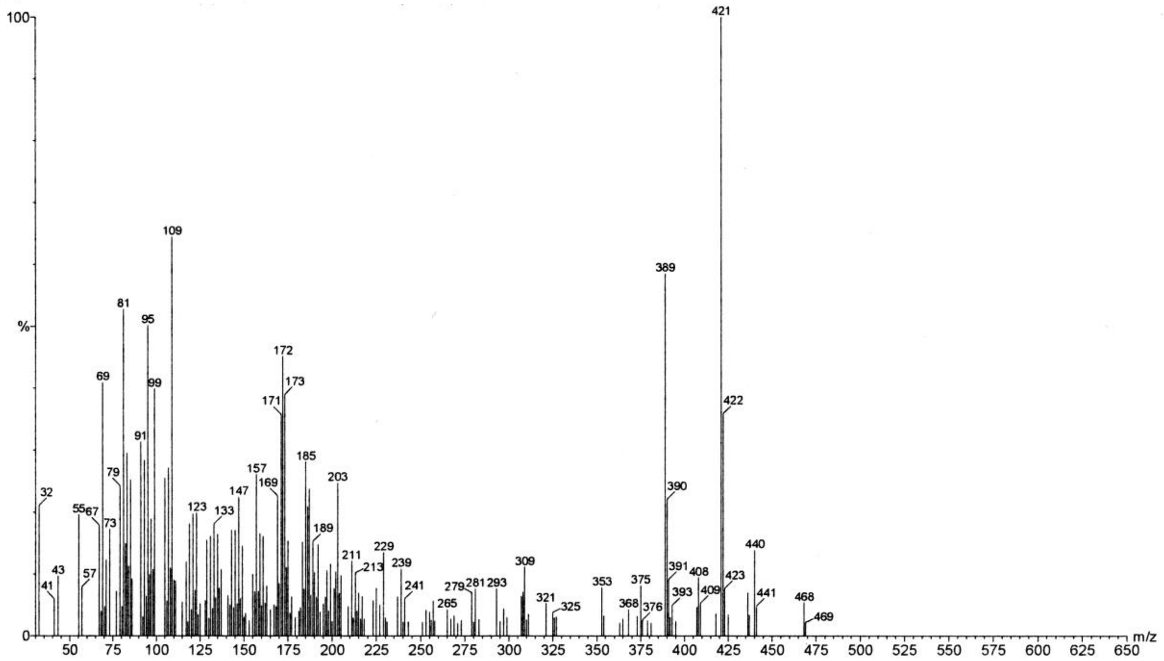

S7. Mass spectrum of DMC
